# Expression of glucagon-like peptide-1 and glucose-dependent insulinotropic polypeptide in the rat submandibular gland is influenced by pre- and post-natal high-fat diet exposure

**DOI:** 10.3389/fphys.2024.1357730

**Published:** 2024-03-26

**Authors:** Pornchanok Sangsuriyothai, Ippei Watari, Saranya Serirukchutarungsee, Sirichom Satrawaha, Katarzyna Anna Podyma-Inoue, Takashi Ono

**Affiliations:** ^1^ Department of Orthodontic Science, Graduate School of Medical and Dental Sciences, Tokyo Medical and Dental University (TMDU), Tokyo, Japan; ^2^ Department of Orthodontics, Faculty of Dentistry, Chulalongkorn University, Bangkok, Thailand; ^3^ Department of Pedodontics and Preventive Dentistry, Faculty of Dentistry, Srinakharinwirot University, Bangkok, Thailand; ^4^ Department of Biochemistry, Graduate School of Medical and Dental Sciences, Tokyo Medical and Dental University (TMDU), Tokyo, Japan

**Keywords:** submandibular gland, GIP, GLP-1, incretins, high-fat diet

## Abstract

**Background:** Incretins, i.e., glucagon-like peptide-1 (GLP-1) and glucose-dependent insulinotropic polypeptide (GIP) promote insulin secretion to reduce postprandial blood sugar. Previous studies found incretins in the salivary glands. However, the role of GLP-1 and GIP in the submandibular gland (SMG) is unclear. This study investigates the effects of a high-fat diet (HFD) on the expression of GLP-1 and GIP throughout the development of rat SMG.

**Methods:** Pregnant 11-week-old Wistar rats were divided into two groups: those fed on a standard diet (*n* = 5) and those fed on a HFD (*n* = 5). From day 7 of pregnancy and throughout the lactation period, all the rats were fed on either a chow diet or HFD. The newborns were divided into four subgroups (*n* = 6): standard diet males (SM), HFD males (HM), standard diet females (SF), and HFD females (HF). The SMGs of 3- and 10-week-old rats from each subgroup were collected under general anesthesia. Moreover, body weight, food intake, and fasting blood sugar were measured. The mRNA expression of GLP-1 and GIP was quantified, and the localization was observed using immunohistochemistry (*p* < 0.05).

**Results:** GLP-1 mRNA expression was statistically significantly more upregulated in HM than in HF at 3 weeks. Moreover, GLP-1 mRNA expression was significantly higher in HM than in both SM and HF at 10 weeks. Although a decreasing trend was observed in GIP mRNA expression in both 3- and 10-week-old rats fed on a HFD, a significant difference between HM and SM only occurred at 3 weeks. Furthermore, the GIP mRNA expression of HM was lower than that of HF at 10 weeks. Immunohistochemical staining revealed GLP-1 and GIP expression mainly in the SMG duct system. Moreover, vacuolated cytoplasm in the duct was observed in rats fed on a HFD.

**Conclusion:** Exposure to HFD during pre- and post-natal periods increased GLP-1 mRNA expression in the SMGs of male rats. However, GIP expression decreased following the HFD in male newborns. Furthermore, a decreasing trend of GIP mRNA expression was observed in male newborns after HFD feeding. Sex influenced incretin hormones secretion and obesity-related conditions. HFD during pre- and post-natal periods reprograms the epigenome, contributing to subsequent disease development.

## 1 Introduction

Maternal nutritional imbalances throughout critical periods of development may have adverse health effects on offspring (Sutton et al., 2016; Fleming et al., 2018). According to the Developmental Origins of Health and Disease (DoHaD) theory, metabolic syndrome is potentially programmed throughout development and early post-natal life by external factors, such as malnutrition, overnutrition, and undernutrition. These exposures also include maternal overweight and obesity (Aiken and Ozanne, 2013; Sutton et al., 2016). Evidence suggests that pre- and post-natal exposure to a high-fat diet (HFD) worsens disease pathogenesis (De Almeida Faria et al., 2017; Tain et al., 2017). Moreover, maternal nutrition throughout the pre- and early post-natal periods is associated with epigenetic mechanisms that play a role in the development of non-communicable diseases, including obesity, metabolic syndrome, and type 2 diabetes (Mendes-Da-Silva et al., 2014; Fleming et al., 2018). Epigenetic changes, obtained throughout pre-natal development or later in life, might impact an individual’s reaction to external stimuli. These changes may explain the differences in the heredity of complicated traits beyond the DNA sequence (Sutton et al., 2016). Therefore, understanding the entire course of disease development is crucial for developing appropriate interventions to prevent non-communicable diseases.

Saliva is essential for preserving oral health and maintaining the functions of the digestive system. The salivary fluid contains enzymes, growth factors, and hormones that are essential for digestion, lubrication, antibacterial defense, and buffering (Kumar et al., 2017; Uchida and Ovitt, 2022). Among the gastrointestinal hormones, the incretin hormones; glucagon-like peptide-1 (GLP-1) and glucose-dependent insulinotropic polypeptide (GIP), are present in saliva (Zolotukhin, 2013). GLP-1 is stimulated by ingesting fats and carbohydrates and stimulates insulin secretion in a glucose concentration-dependent manner (Baggio and Drucker, 2007; Chia and Egan, 2020; Müller et al., 2019). Additionally, GLP-1 can inhibit glucagon secretion (Baggio and Drucker, 2007; Chia and Egan, 2020). GLP-1 has also been found in the taste cells of the taste buds and salivary glands of rodents (Feng et al., 2012; Ono et al., 2015). GIP is secreted in response to nutrients, such as glucose, fat, and protein to potentiate glucose-stimulated insulin secretion in a dose-dependent manner (Chia and Egan, 2020; Baggio and Drucker, 2007). Compared with glucose, fat is a more effective GIP secretagogue (Chia and Egan, 2020). Although GIP has been found in the stomach and duodenum of both rodents and humans, GIP expression was exclusively investigated in the salivary gland of rats (Tseng et al., 1995; Baggio and Drucker, 2007; Privatananupunt et al., 2014). Moreover, GIP was also detected in human saliva (Messenger et al., 2003).

The influence of sex and HFD on GLP-1 and GIP expression is unresolved. A prior study demonstrated that GLP-1 concentration was not influenced by human characteristics, including age and sex (Nauck, 2004). In contrast, several studies have found that sex influences GLP-1 (Kautzky-Willer et al., 2016; Santos-Marcos et al., 2018). Although a study on GIP in the saliva and plasma of humans was conducted, the function of GIP remains unclear (Messenger et al., 2003). Currently, limited studies have reported the expression of incretin hormones in the salivary gland. However, their exact functions and biological roles are unknown. Accordingly, the aim of this study is to investigate the expression of GLP-1 and GIP in the submandibular gland (SMG) of rats under the HFD condition from the onset of salivary gland development. Furthermore, we investigate the impact of sex on the response of GLP-1 and GIP in SMG throughout fetal development and feeding in the post-natal period with a HFD.

## 2 Materials and methods

### 2.1 Animals and experimental design

**FIGURE 1:**
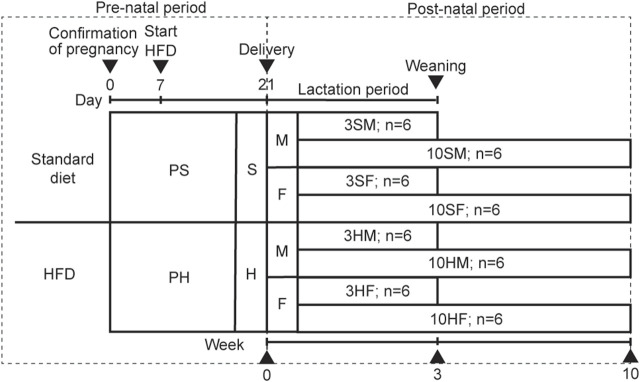
Animal experimental design. Ten pregnant Wistar rats, aged 11 weeks old, were divided equally into two groups: PS, which was fed on a standard diet; PH, which was fed on a HFD. The offspring from both groups were equally divided into four groups based on their mothers’ diet: standard males (SM), standard females (SF), HFD males (HM), and HFD females (HF). The HFD was given to the designated groups starting on day 7 of pregnancy until the rats reached 3 weeks or 10 weeks of age. The 3-week-old rats were sacrificed at the end of the weaning period (3SM, 3SF, 3HM, and 3HF), followed by the 10-week-old rats (10SM, 10SF, 10HM, and 10HF). HFD, high-fat diet.

Ten pregnant 11-week-old Wistar rats (Sankyo Labo Service Corporation, Tokyo, Japan) were raised separately in their containers in a humidity-controlled room under a 12-h light–dark cycle and a controlled temperature of 23°C. The rats were randomly fed equally either on an *ad libitum* standard diet or HFD. The pregnant rats were divided into two groups based on the feed: a standard diet (pregnant standard diet [PS] group) or a HFD (pregnant HFD [PH] group). The food type, amount, and percentage of fat in each of the PS and PH groups were: CE-2 (Clea, Tokyo, Japan), 340.2 kcal/100 g, 4.6% of fat; and HFD32 (Clea, Tokyo, Japan), 507.6 kcal/100 g, 32% of fat, respectively. The source of fat in CE-2 was derived from cereal germ and soybean oil. In contrast, the source of fat in HFD32 was derived from powdered beef tallow and safflower oil (Supplementary Table S1). Because the peak sensitivity for the embryo’s development is in the second trimester and before the salivary gland was established in the late second trimester, the specific diet of the pregnant rats was commenced on day 7 of pregnancy (Quirós-Terrón et al., 2019; Damen et al., 2021; Harada et al., 2023). The diet was also continued during the breastfeeding period. The male and female PS offspring were categorized as the 3SM (3-week-old, male, *n* = 6), 10SM (10-week-old, male, *n* = 6), 3SF (3-week-old, female, *n* = 6), and 10SF (10-week-old, female, *n* = 6) groups. The other male and female offspring in the PH group were categorized as 3HM (3-week-old, male, *n* = 6), 10HM (10-week-old, male, *n* = 6), 3HF (3-week-old, female, *n* = 6), and 10HF (10-week-old, female, *n* = 6) groups. After weaning, the pups continued consuming the same diet as their mothers to ensure that the nutrition environment was identical to that of their mothers (Figure 1). Animal protocols were approved by The Institutional Animal Care and Use Committee of Tokyo Medical and Dental University (A2020-148A). The animal experiments were performed in accordance with the University’s Guidelines for Animal Experimentation.

### 2.2 Mother rat weight, food intake, and fasting blood sugar level

The body weight (BW) and food intake of each mother rat were determined daily. Food consumption was calculated by subtracting the measurement of the leftover food from that of the food before consumption. To measure the fasting blood sugar (FBS) levels using a glucometer (Accu-Chek guide, Roche, Basel, Switzerland), the rats were fasted for 8 h, and blood was obtained from the tail vein under inhaled anesthesia (Isoflurane, Wako, Osaka, Japan). The mother rats were sacrificed at the age of 17 weeks.

### 2.3 Offspring weight, food intake, and fasting blood sugar level

The offspring rats’ BW and food consumption were measured daily. Food intake was calculated using the same method as for the mother rats. To measure the FBS levels using a glucometer, the rats were fasted for 8 h, and blood was obtained from the tail vein under inhaled anesthesia. The offspring rats were sacrificed at the age of 3 and 10 weeks. The capsulated SMG of each rat at 3- and 10-week-old was isolated under general anesthesia and weighed immediately (wet weight) according to the protocol mentioned in previous studies (Privatananupunt et al., 2014; Ono et al., 2015). The right SMG was collected into Sepasol^®^-RNA I Super G (Nacalai Tesque, Inc, Kyoto, Japan) for isolating of the total RNA and reverse transcription-quantitative polymerase chain reaction (RT-qPCR). The left SMG was collected for immunohistochemical staining.

### 2.4 Immunohistochemical staining

Each offspring’s left SMG was fixed in 4% formaldehyde (Mildform 10 NM, Wako, Osaka, Japan) for 24 h before being embedded in paraffin according to the previous protocols (Privatananupunt et al., 2014; Ono et al., 2015). The paraffin-embedded SMG was sliced into 5-µm-thick sections (Leica, HistoCore AUTOCUT R, Wetzlar, Germany) and stained with either GLP-1 or GIP antibodies to evaluate their expression. To assess the specificity of the immunohistochemical staining and identify false-positive staining responses, the negative controls were not incubated with primary antibodies.

The specimens prepared for GLP-1 staining were deparaffinized with xylene and rehydrated in the descending ethanol concentrations. Antigen retrieval was performed using a microwave-heated sodium citrate buffer (pH 6.0). The sections were incubated for 20 min and cooled down at room temperature. After rinsing with phosphate-buffered saline with Tween^®^ 20 (PBST), the endogenous peroxidase activity was inhibited by covering the sections with 3% H_2_O_2_ in methanol for 15 min at room temperature. The slides were carefully washed with PBST before being incubated with diluted normal-blocking serum for 20 min. The samples were then incubated overnight in a humidified chamber at 4°C with GLP-1 (ab111125, Abcam, Cambridge, United Kingdom) diluted in PBS containing 0.1% bovine serum albumin (BSA). After rinsing with PBST, the sections were incubated with a diluted biotinylated antibody (VECTASTAIN^®^ Elite ABC-HRP Kit, PK-6101, Vector Laboratories, CA, United States) for 30 min at room temperature, followed by the incubation of VECTASTAIN Elite ABC Reagent for 30 min at room temperature.

To localize the GIP expression, the processes of deparaffinization, rehydration, and antigen retrieval followed the GLP-1 protocol. The sections were rinsed in PBS and incubated with GIP antibody (ab25973, Abcam, Cambridge, United Kingdom). The primary antibody was diluted in PBS containing 1% BSA.

To visualize the immunoreactivity of GLP-1 and GIP, 3,3′-diaminobenzidine (DAB) (ab64238, Abcam, Cambridge, United Kingdom) was applied, followed by Mayer’s hematoxylin (Wako, Osaka, Japan) for counterstaining. The sections were mounted using Mount-Quick (Daido Sangyo, Saitama, Japan). The images were obtained using a light microscope (Nikon, Eclipse 80i, Tokyo, Japan) equipped with a digital camera (Nikon, DS-Ri1, Tokyo, Japan). The images were standardized before processing.

The semi-quantitative immunohistochemical analysis of GLP-1 and GIP expression in SMGs was performed using the imaging software as previously described (Fagundes et al., 2016). Also, the optical density of immunoreactive areas in SMG was quantified using ImageJ software (ImageJ2 v2.14.0/1.54f, NIH, United States). In brief, five randomly selected regions for each sample at a magnification of ×400 were acquired with a microscope connected to a digital camera. The “color deconvolution plug-in” from ImageJ was used to segment and separate the DAB-stained areas (Landini et al., 2021). Following the segmentation of the image, the optical density was measured.

### 2.5 RT-qPCR

Total RNA was extracted from SMGs using Sepasol-RNA I Super G. The protocols were as follows: first, the homogenized samples were kept at room temperature in Sepasol-RNA I Super G for 5 min. Then, 200 μL chloroform was added and mixed well. After 3-min incubation at room temperature, the tubes were centrifuged at 12,000 × g for 15 min at 4°C. The supernatant was collected and mixed with 500 μL 2-propanol. After 10 min at room temperature, the tubes were centrifuged at 12,000 × g for 10 min at 4°C. The supernatant was removed by centrifugation at 12,000 × g for 5 min at 4°C, and the RNA pellet was washed with 1,000 μL ethanol. The quality of total RNA was evaluated using a spectrophotometer at 260 nm. RNA was kept at −80°C until usage.

The mRNA expression levels of GLP-1 and GIP in SMG were analyzed using RT-qPCR. The reaction was performed by synthesizing complementary DNA (cDNA) using ReverTra Ace^®^ qPCR RT Master Mix (Toyobo, Osaka, Japan) from 1 μg total RNA. Subsequently, RT-qPCR was performed using the StepOne™ Real-Time PCR System (Applied Biosystems, CA, United States). The 20 µL reaction mixture contained cDNA template, probe qPCR Mix (Takara, Shiga, Japan), TaqMan™ primer, probe, and ROX reference dye. The TaqMan™ primer-probe sets were GAPDH (Rn99999916_s1), GLP-1 (Rn00562293_m1), and GIP (Rn00571500_m1). The TaqMan™ assays were purchased from Thermo Fisher Scientific (MA, United States). The thermal conditions followed the manufacturer’s recommendations. Briefly, the reaction conditions were: 95°C for 20 s, 40 cycles at 95°C for 1 s, and 60°C for 20 s. The average expression of GLP-1 and GIP in SMG in each group was calculated using the threshold cycle (Ct) value and compared using StepOne™ Software v2.2.2 (Applied Biosystems). The relative quantification (RQ) method was used to investigate the fold difference in mRNA expression. The endogenous control was GAPDH, and all samples were quantified in triplicate. The 2^−ΔΔCT^ formula was used for calculating RQ. To compare the gene expression between groups, the relative expression value for each gene was used.

### 2.6 Statistical analysis

The sample size was calculated using the mean and standard deviation from the pilot study. Based on the sample size calculated using G*Power software (version 3.1.9.6, Kiel University, Kiel, Germany), the number of rats required per group to ensure a sufficient power of 80% was *n* = 6. The Shapiro–Wilk test was used to check for the normal distribution of the mother rats’ data (average energy intake and fat intake), followed by an unpaired t-test. To compare the weight and FBS levels between groups, a two-way analysis of variance (ANOVA) was performed, followed by Fisher’s least significant difference (LSD). The offspring’s physiological data were compared among the four groups. For multiple comparisons of sex and feeding type, the two-way ANOVA was performed, followed by Fisher’s LSD in case of a statistically significant difference. *p* < 0.05 was considered statistically significant. Data were reported as mean ± standard error of the mean (SEM). All data were analyzed using GraphPad Prism software (GraphPad version 10.0.2, GraphPad Software, CA, United States).

## 3 Results

### 3.1 Effects of the HFD on the PS and PH groups characteristics

**FIGURE 2:**
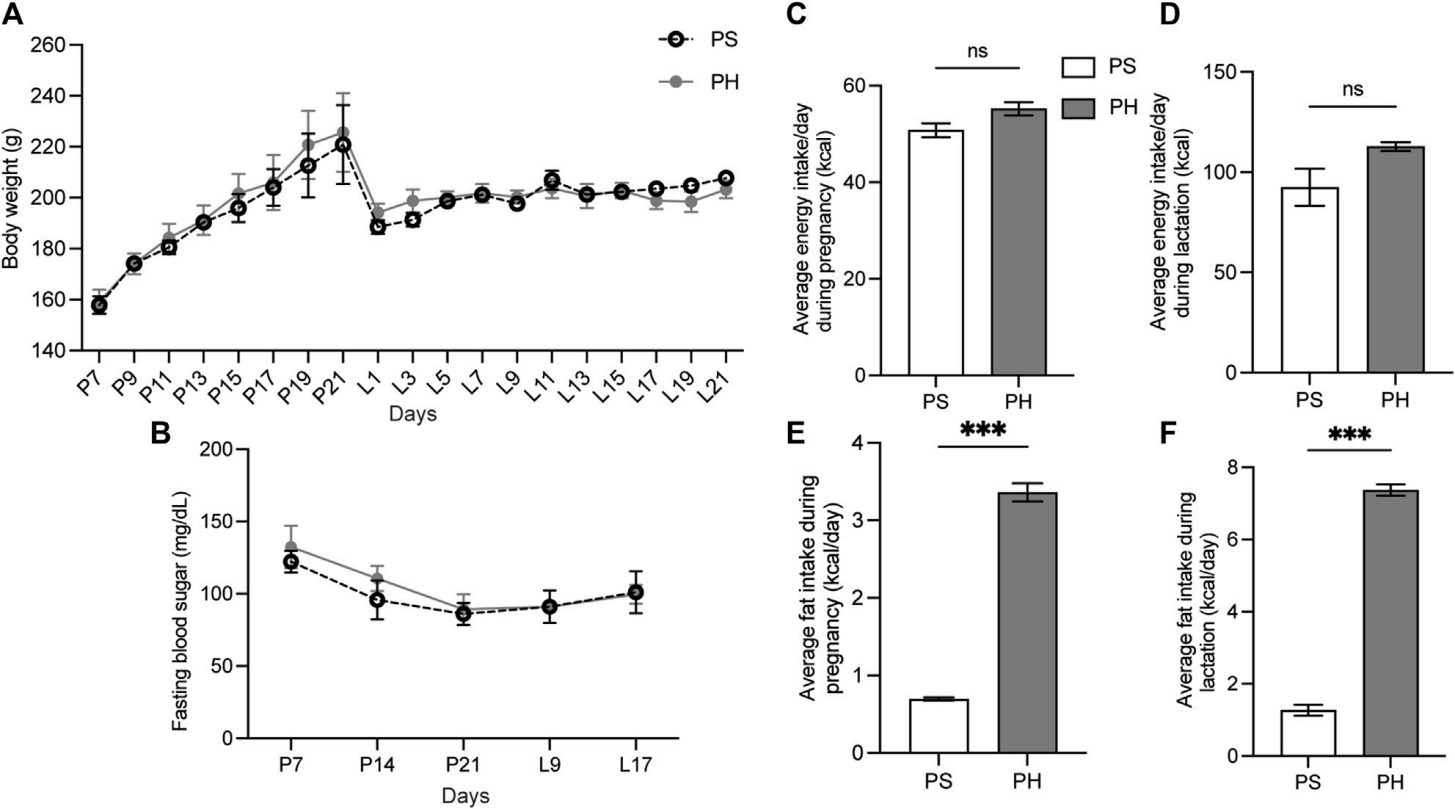
The physiological data of the mother rats. The mother rats were not significantly affected by HFD. The BW of PS and PH rats remained comparable throughout the experiment **(A)**. PS and PH groups had similar fasting blood sugar levels **(B)**. Energy consumption during pregnancy and breastfeeding was comparable between PS and PH **(C, D)**. However, PH rats consumed more energy from fat compared with the PS rats throughout both pregnancy **(E)** and lactation periods **(F)**. The data are expressed as mean ± SEM (***: *p* < 0.001, ns: not significant). HFD, high-fat diet; BW, body weight; PS, pregnant rats fed on a standard diet; PH, pregnant rats fed on a high-fat diet; SEM, standard error of the mean.

The HFD was administered to mother rats on the seventh day of pregnancy to ensure that the HFD covered the entire development of the SMGs. However, the BW of rats in both PS and PH groups did not significantly differ during the experimental period (Figure 2A). The FBS levels in the rats in both PS and PH groups were comparable (Figure 2B). The amount of energy consumed by PS and PH throughout pregnancy and lactation was comparable (Figures 2C, D). However, PH rats consumed more energy from fat compared with the PS rats throughout the pregnancy (Figure 2E) and lactation periods (Figure 2F).

### 3.2 Effects of the HFD on the characteristics of the rats among different offspring groups

**FIGURE 3:**
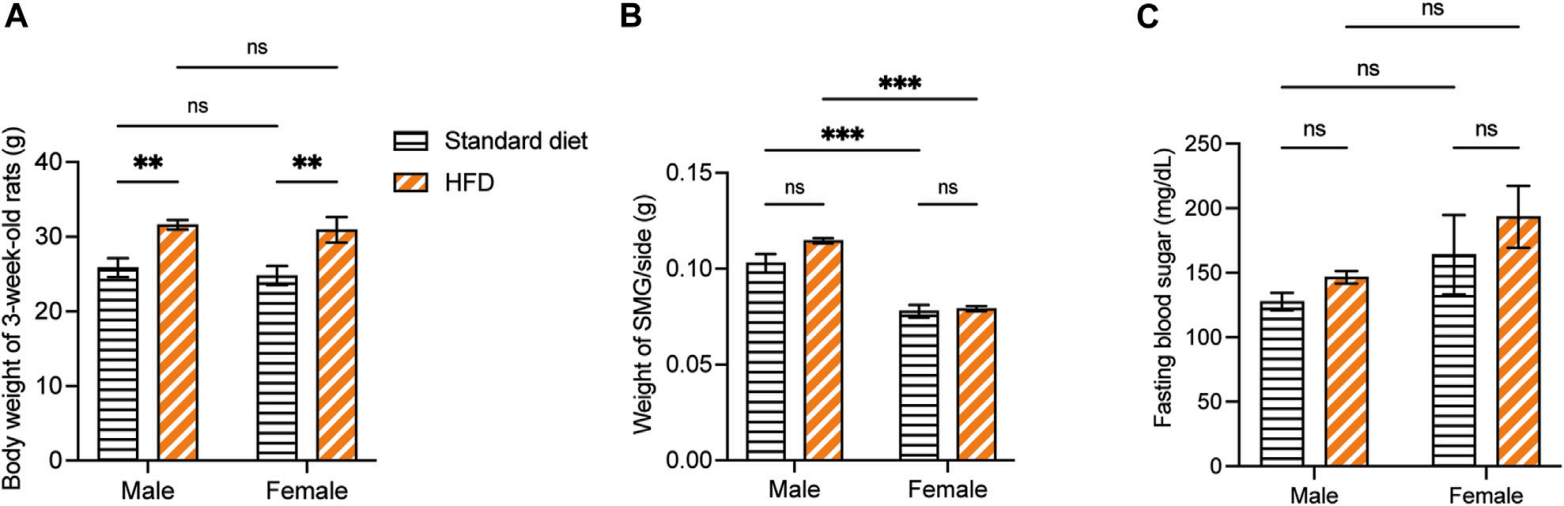
The physiological data of 3-week-old rats. The BW of 3HM and 3HF was affected by HFD. Specifically, the BW of 3HM and 3HF was greater than that of 3SM and 3SF respectively **(A)**. The wet weight of SMG was heavier in male rats than in female rats **(B)**. However, there was no difference in FBS among the different groups of 3-week-old rats **(C)**. The data are expressed as mean ± SEM (**: *p* < 0.01, ***: *p* < 0.001, ns: not significant). BW, body weight; HM, HFD males; HF, HFD females; HFD, high-fat diet; SM, standard males; SF, standard females; SMG, submandibular gland; FBS, fasting blood sugar; SEM, standard error of the mean.

**FIGURE 4:**
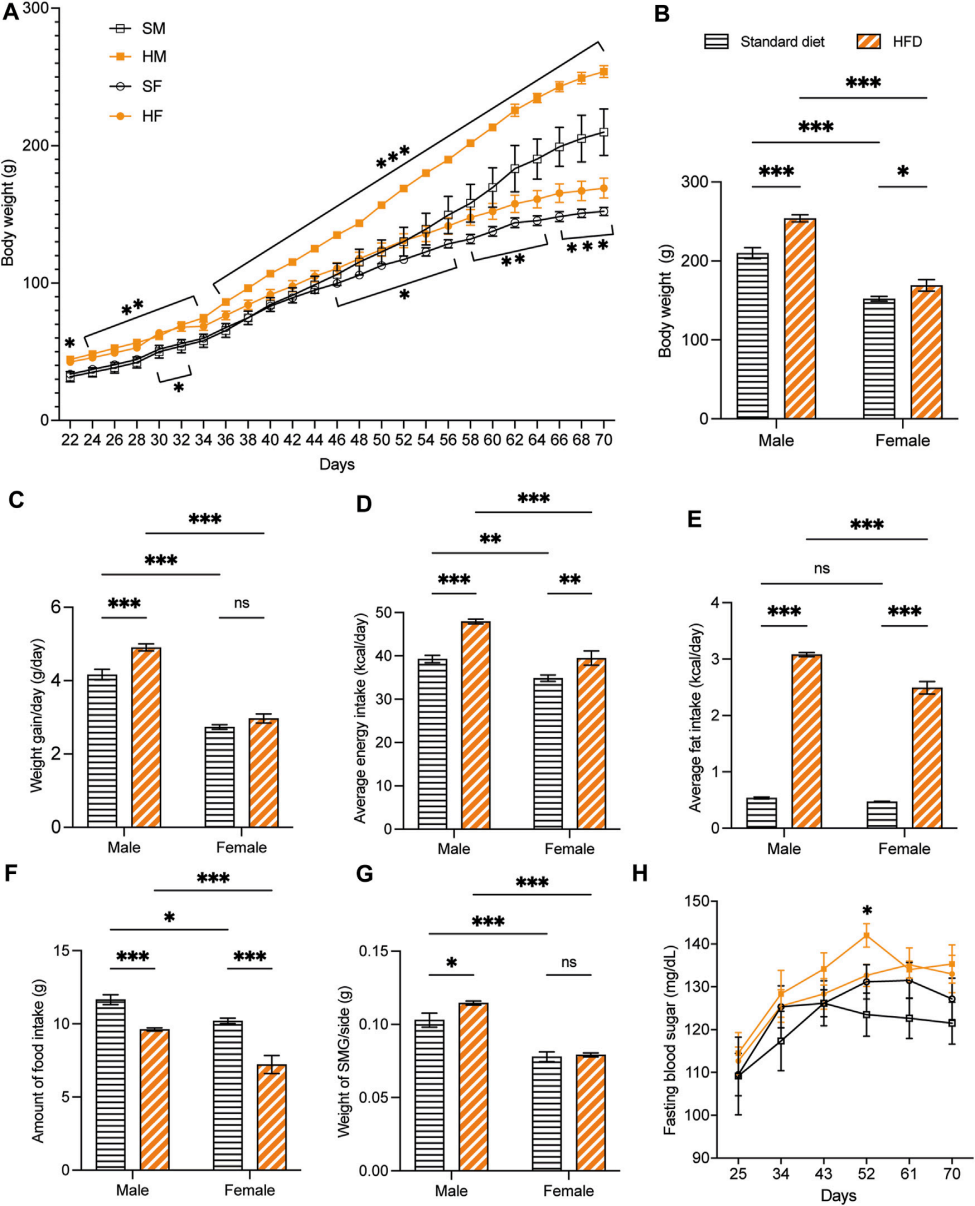
The physiological data of 10-week-old rats. HFD-fed male rats showed more alterations after being fed on a HFD. The BW of 10HM and 10HF were heavier than those of 10SM and 10SF, respectively **(A, B)**. The average weight increase per day in 10HM was larger than in 10SM, but there was no difference between female groups **(C)**. Both male and female rats in HFD-fed groups consumed more daily calories and fat than their control counterparts **(D, E)**. However, the amount of food consumed was significantly reduced in HFD-fed rats **(F)**. The wet weight of SMG per side was heavier in 10HM than in 10SM **(G)**. FBS level was significantly higher in 10HM than in 10SM at 52 days of age **(H)**. The data are expressed as mean ± SEM (*: *p* < 0.05, **: *p* < 0.01, ***: *p* < 0.001, ns: not significant). HFD, high-fat diet; BW, body weight; HM, HFD males; HF, HFD females; SM, standard males; SF, standard females; SMG, submandibular gland; FBS, fasting blood sugar; SEM, standard error of the mean.

Overall, the weaning BW of the 3-week-old rats was affected by the HFD in both sexes. The BW of 3HM and 3HF was significantly greater than those in the matched control diet groups, 3SM and 3SF (Figure 3A). The SMGs’ wet weight in the 3SM and 3HM groups was significantly heavier than those of the 3SF and 3HF groups (Figure 3B). No significant difference was observed in the FBS of the 3-week-old rats among the groups (Figure 3C). In contrast, an interaction effect of food and sex was observed on the BW in 10-week-old rats. The BW of the 10HM group was greater than that of the 10SM and 10HF groups (Figures 4A, B). Additionally, the interaction effect of sex and diet was also observed on weight gain per day. Male rats gained more weight than females (Figure 4C). Moreover, a difference was found in the average daily energy intake between male and female rats on standard diets. The mean energy consumption in the 10HM and 10HF groups was significantly greater than that of the 10SM and 10SF groups (Figure 4D). No significant difference was observed in the energy from fat consumption between male and female standard diet-fed rats. However, the amount of fat consumed by the 10HM and 10HF rats was greater than that of the 10SM and 10SF rats. Additionally, the amount of fat intake among males and females was significantly different between the 10HM and 10HF groups. Hence, the interaction between sex and food was also illustrated (Figure 4E). Furthermore, the 10HM and 10HF groups consumed significantly less food compared with the 10SM and 10SF groups (Figure 4F). The wet weights of SMGs in the 10SM and 10HM groups were heavier than those in the 10SF and 10HF. The wet weight of SMG in male rats was greater than that of female rats, regardless of whether the groups were fed on a standard diet or HFD. Furthermore, the wet weight of SMGs increased significantly only in males following HFD compared to that in the 10SM group (Figure 4G). In the 10-week-old rats, although the FBS levels were higher in the HFD groups than in the standard diet groups at every time point, the difference was not significant except at 52 days of age between HM and SM (Figure 4H).

### 3.3 Localization and expression of GLP-1 and GIP in the SMG of offspring rats

**FIGURE 5:**
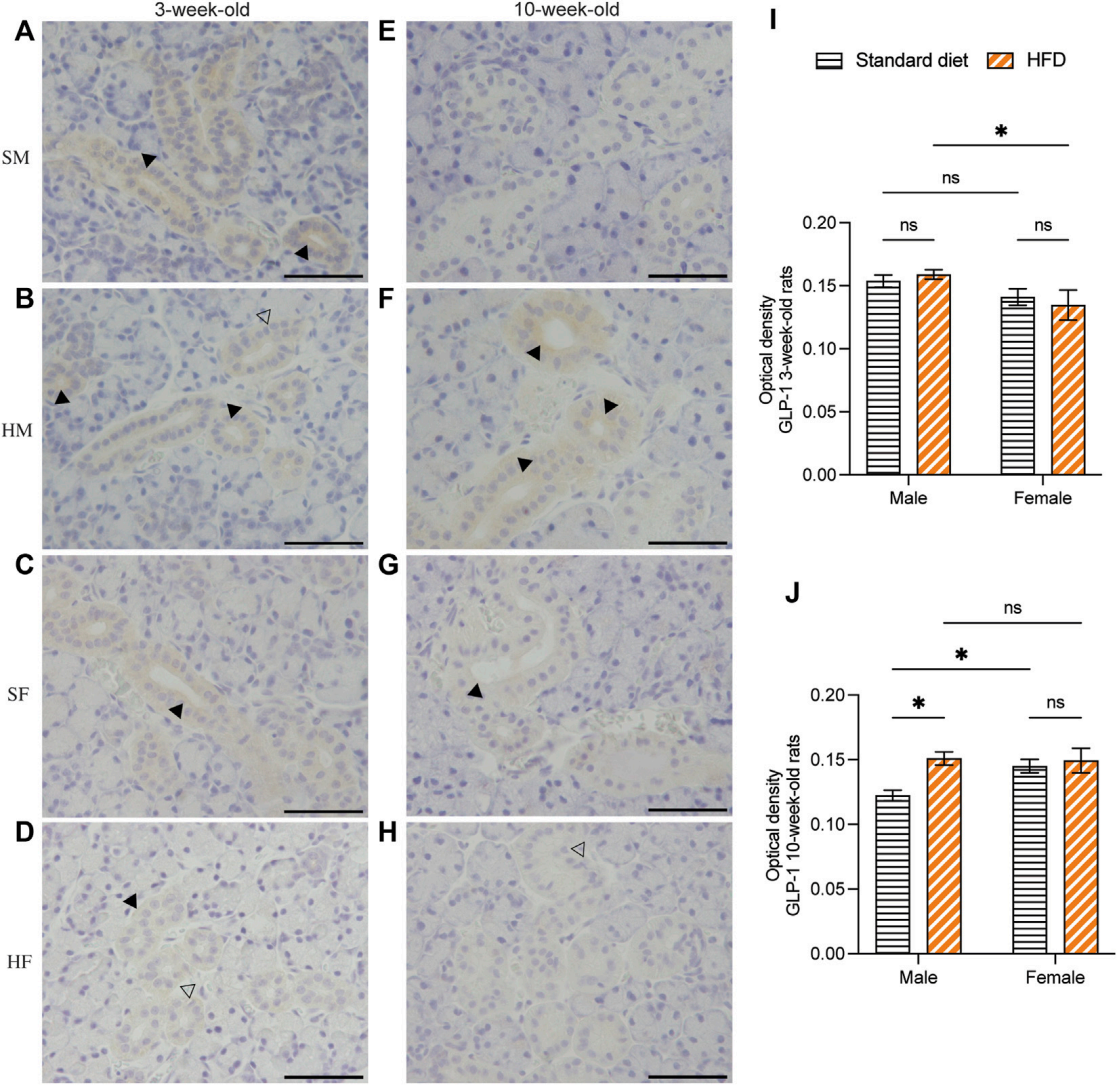
Immunohistochemical staining of GLP-1 in 3- and 10-week-old rats. Immunohistochemical staining of GLP-1 in the SMGs of 3-week-old rats **(A–D)**. Immunohistochemical staining of GLP-1 in the SMGs of 10-week-old rats **(E–H)**. Scale bars = 100 μm. Semi-quantitative immunohistochemical analysis of GLP-1 in the SMGs of 3- and 10-week-old rats showed significantly higher immunoreactivity in 3HM than in 3HF **(I)**. Additionally, the levels of GLP-1 were observed to be lower in 10SM when compared to both 10HM and 10SF **(J)**. The black arrows indicate increased intensity of GLP-1 at the area contacting the lumen of the duct, while the clear arrows indicate the presence of intra-cytoplasmic vacuolations identified in the HFD groups. The data are expressed as mean ± SEM (*: *p* < 0.05, ns: not significant). GLP-1, glucagon-like peptide-1; SMG, submandibular gland; HM, HFD males; HF, HFD females; SM, standard males; SF, standard females; HFD, high-fat diet; SEM, standard error of the mean.

**FIGURE 6:**
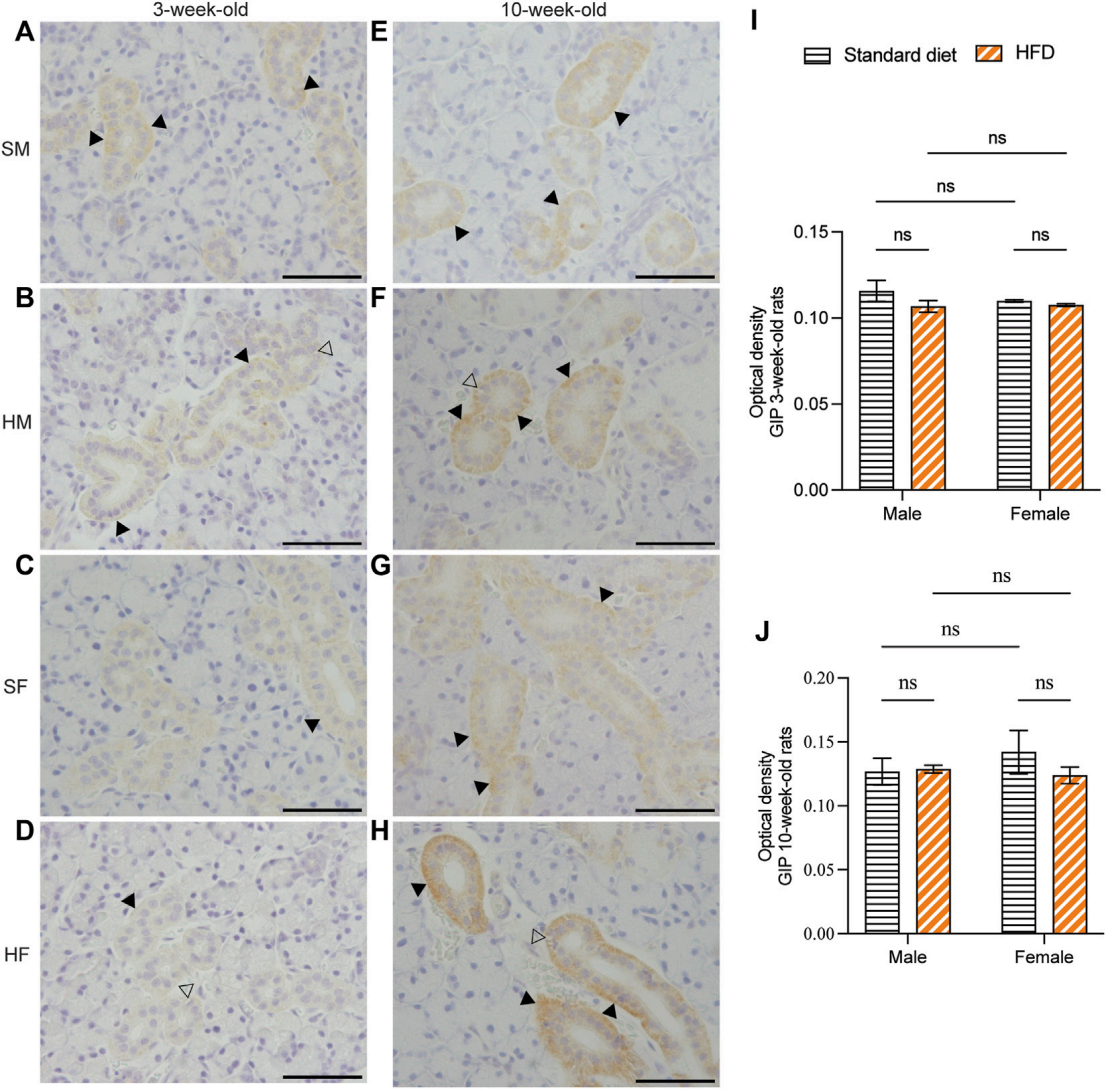
Immunohistochemical staining of GIP in 3- and 10-week-old rats. Immunohistochemical staining of GIP in the SMGs of 3-week-old rats **(A–D)**. Immunohistochemical staining of GIP in the SMGs of 10-week-old rats **(E–H)**. Scale bars = 100 μm. Semi-quantitative immunohistochemical analysis of GIP in the SMGs of 3- and 10-week-old rats revealed no statistically significant differences between the groups **(I, J)**. The black arrows indicate increased intensity of GIP at the striation of the striated duct, while the clear arrows indicate the presence of intra-cytoplasmic vacuolations identified in HFD groups. The data are expressed as mean ± SEM (ns: not significant). GIP, glucose-dependent insulinotropic polypeptide; SMG, submandibular gland; HFD, high-fat diet; SEM, standard error of the mean.

Immunohistochemical staining of GLP-1 and GIP revealed a comparable structure of the duct in every group. However, vacuolated cytoplasm was observed in rats fed on a HFD. The GLP-1 and GIP immunohistochemical staining was detected in the duct systems of the rats’ salivary glands. The staining areas were mainly observed in the epithelial cell cytoplasm of the intercalated, granular, striated, and excretory ducts. The immunostaining was not detected in acinar cells. GLP-1 and GIP expression was not detected in the negative controls (data not shown). Immunoreactivity for GLP-1 were uniformly distributed throughout the ductal areas. The intensity increased in the areas in contact with the lumen of the duct (Figures 5A–H). Semi-quantitative analysis of GLP-1 revealed more intense immunoreactivity in the 3HM compared with that in the 3HF (Figure 5I). Compared with the 10HM, the 10SM demonstrated reduced immunoreactivity. Moreover, an increased staining intensity was observed in 10SF compared with 10SM (Figure 5J). In contrast, the GIP immunoreactivity was spread across the ductal area. Furthermore, we also observed a greater intensity in the striations of the striated ducts (Figures 6A–H). No significant differences were observed between the groups in the semi-quantitative analysis of GIP immunostaining (Figures 6I, J).

### 3.4 Effects of HFD on GLP-1 and GIP mRNA expression in the SMG of offspring rats

**FIGURE 7:**
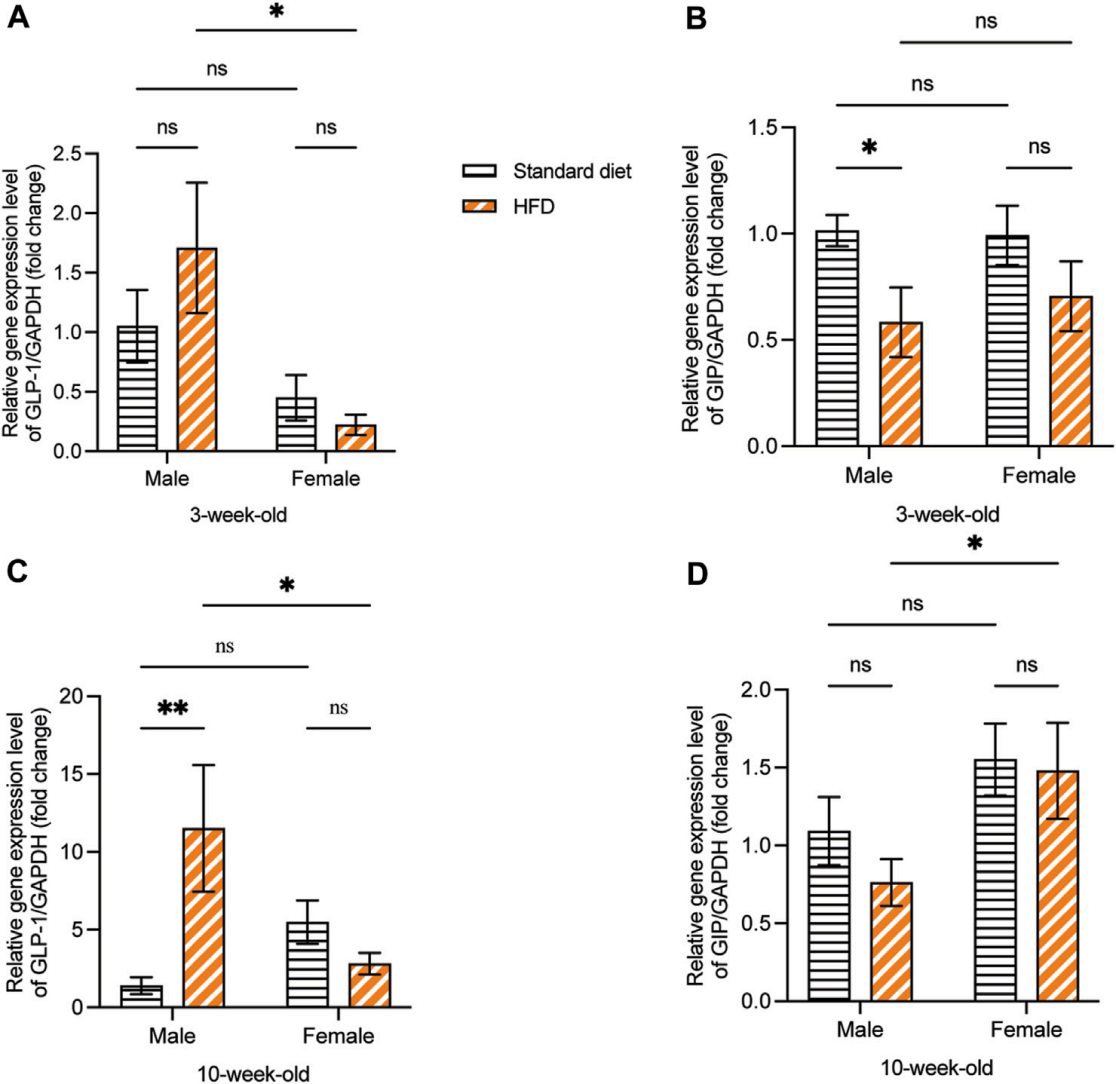
The mRNA expression of GLP-1 and GIP. HFD-fed male rats showed an increased mRNA expression of GLP-1 and a decreased mRNA expression of GIP. GLP-1 mRNA expression was significantly higher in 3HM than in 3HF **(A)**. GIP mRNA expression in 3HM was significantly lower than that in 3SM **(B)**. GLP-1 mRNA expression was significantly higher in 10HM than in 10SM and 10HF **(C)**. The GIP mRNA expression in 10HF was higher than that in 10HM **(D)**. Data are expressed as mean ± SEM (*: *p* < 0.05, **: *p* < 0.01, ns: not significant). GLP-1, glucagon-like peptide-1; GIP, glucose-dependent insulinotropic polypeptide; HM, HFD males; HF, HFD females; SM, standard males; SEM, standard error of the mean.

At 3 weeks, although no significant difference was observed, the GLP-1 mRNA expression showed a higher trend in 3HM compared with 3SM. Moreover, no significant difference was observed between the 3SF and 3HF groups. Compared with female rats fed on a HFD, male rats had significantly higher GLP-1 mRNA expression (Figure 7A). The relative GIP mRNA expression in offspring rats in the 3HM group was significantly downregulated compared with that in the 3SM group. However, no significant difference was observed between the 3SF and 3HF groups (Figure 7B). In 10-week-old rats, the GLP-1 mRNA level was significantly higher in the 10HM compared with the 10SM. However, no significant difference was observed between 10SF and 10HF. The interaction between sex and diet was observed in 10-week-old rats (Figure 7C). In contrast, a significant difference was observed in GIP mRNA expression between 10HM and 10HF. The GIP mRNA expression level in the 10HF group was significantly higher than that in the 10HM, whereas no significant difference was observed between 10SM and 10SF (Figure 7D).

## 4 Discussion

### 4.1 Short-term effects of HFD during pre- and post-natal periods on offspring rats’ characteristics

Although minor disparities were observed in the FBS levels and BW between the PS and PH groups during pregnancy and breastfeeding periods, no significant differences were observed. The PH group consumed significantly more calories from fat than the PS group during pregnancy and lactation. However, the mean energy intake was comparable to that of the PS group. The development of SMG in a rodent embryo also begins approximately at the late second trimester and completes at the seventh to tenth week after birth (Bano and Ghafoor, 2018; Harada et al., 2023). In our study, the HFD groups commenced HFD at the beginning of the second trimester. A hostile intrauterine environment also induces epigenetic programming, which is crucial for the differentiation and development of embryos (Fleming et al., 2018). Notably, the outcome worsened in rats if offspring whose mothers were HFD-fed during pregnancy continued eating the high-energy diet after weaning (Krasnow et al., 2011; Serirukchutarungsee et al., 2023). Moreover, the timing of the intrauterine exposure might cause diverse programming consequences (Sutton et al., 2016). In humans, the second trimester of pregnancy is critical for the development of the fetus (Damen et al., 2021). Therefore, the relationship between fat consumption and the baby’s body fat percentage during this time was the strongest (Catalano and Shankar, 2017; Damen et al., 2021). The lipid in the mother’s circulation changes the composition of fat in the human fetus (Szabo, 2019). Moreover, due to the mothers’ excessive fat diet, the amount of fatty acid reaching the developing human fetus through the placenta increases (Damen et al., 2021; Szabo, 2019). In our study, the 3-week-old rats primarily received nutrients via the placenta and milk. Furthermore, the HFD-fed mother rats had higher levels of fatty acid and leptin in milk than the control diet-fed mothers (Bautista et al., 2016; Tellechea et al., 2017). According to developmental programming in humans and animals, male and female infants had different phenotypes following various intrauterine insults (Aiken and Ozanne, 2013). In animals, the males’ placentas were smaller and consumed more nutrients than the females’ in response to the mother’s hormones (Mao et al., 2010; Ribaroff et al., 2017). Moreover, exposure to overnutrition has been shown to cause developmental programming of offspring metabolism (Fleming et al., 2018; Hagemann et al., 2021). Detrimental intrauterine and early life exposures during organ and system development might result in persistent structural disruptions in the fetus, contributing to the development of chronic conditions later in life (Sutton et al., 2016; Fleming et al., 2018). Therefore, this explanation, along with sex predilection, might contribute to the significantly higher GLP-1 mRNA levels during the intrauterine and lactation periods in the 3HM compared to the 3HF. The GIP level was significantly lower in the 3HM compared with the 3SM. Though not significant, a decreasing trend of GIP expression was observed in the SMG of 3HF compared with 3SF. According to a study in humans, fasting GIP levels in saliva are substantially greater than in plasma. However, the amount in the saliva is dramatically reduced during food ingestion or when sham-fed meals are provided. This process, aimed at minimizing the suppression effect of gastric acid, is intended to improve food digestion and absorption (Messenger et al., 2003). The decreased level of GIP expression observed in the 3HM rats in our study could be due to a similar mechanism observed in humans in the previous study.

### 4.2 HFD increased GLP-1 mRNA expression in 10HM

We found that HFD significantly increased the expression of GLP-1 in 10HM compared with 10SM. In our prescription, HFD contained higher energy (507.6 kcal/100 g) compared to that in the standard diet (340.2 kcal/100 g). The daily energy intake of the 10HM group was significantly higher (47.94 kcal/day) than that of the 10SM (39.29 kcal/day). A previous study revealed that male Sprague–Dawley rats released more GLP-1 after being fed higher-calorie meals (Wang et al., 2015). Although pre-natal insults can significantly affect organ systems in programming offspring, post-natal modification can have activating effects on disease development, expression, or severity (Padmanabhan et al., 2016). Therefore, the increased GLP-1 expression in male rats in our study could be due to the exposure to overnutrition during the intrauterine phase, and the exposure to excessive calorie intake during the post-natal period. In contrast, no significant difference was found in GLP-1 mRNA expression between the 10HF and 10SF groups. Although HFD contained more calories than the standard diet, 10HF consumed less HFD per day (7.21 g/day) compared with the 10HM (9.62 g/day), 10SF (10.21 g/day), and 10SM (11.66 g/day) groups. Therefore, the resultant caloric consumption between 10HF (39.49 kcal/day) and 10SF (34.85 kcal/day) was comparable. Additionally, the daily weight increases were similar for both groups. In contrast, the 10HM BW was significantly greater than that of 10SM and 10HF. Notably, the link between body mass and GLP-1 concentrations in the plasma of humans has been reported (Stinson et al., 2021). Conversely, prior studies in rodents found that GLP-1 levels were reduced after being induced by HFD due to leptin resistance in mice and L-cell dysfunction in rats (Anini and Brubaker, 2003; Zheng et al., 2017).

The lower proportions of HFD consumption in the 10HF and 10HM groups compared to those of the control groups were consistent with the findings of the study indicating that fat dramatically reduces energy intake and appetite in mice (Little and Feinle-Bisset, 2011). According to a previous study, HFD consumption induces the production of oleoylethanolamine (OEA), which mediates the signals of the presence of fat in the small intestine to the central nervous system (Little and Feinle-Bisset, 2011). In our study, the HFD consisted of mono-unsaturated fat, including 64.3% oleic acid. The interaction of oleic acid with the cluster of differentiation 36, known as platelet glycoprotein 4, in the small intestine results in OEA production, which results in a decrease in overall food intake and BW in study mice (Schwartz et al., 2008; Little and Feinle-Bisset, 2011). Moreover, oleic acid influences the expression of genes involved in lipid absorption, lipid synthesis, and glucose pathways (Chen et al., 2018). Similarly, in a previous study in which rats received 40% of their calories from fat, the HFD did not affect blood glucose levels (Reed et al., 2000). Earlier findings showed that foods containing high saturated fat led to more hyperinsulinemia compared to normal and polyunsaturated fat diets in animal models (Lee et al., 2006; Chacińska et al., 2019). Meanwhile, diets with unsaturated fat resulted in decreased plasma insulin concentration and improved insulin sensitivity (Lee et al., 2006; Chacińska et al., 2019; Lang et al., 2019).

Notably, 10HM rats had significantly higher GLP-1 mRNA expression in SMG than the 10HF rats. Female rats achieved control of their energy intake more effectively than males, possibly due to their greater capacity to compensate for the increased calorie density of the diet (Estrany et al., 2013). Furthermore, female rats exhibited a delay in diet-induced weight gain and had fewer metabolic complications than males (Maric et al., 2022). By eliminating the effect of ovarian hormones, an increase in food intake and a decrease in motor activity were observed in rats (Richard et al., 2017). Conversely, another study found that although HFD-fed female rats consumed more energy, they utilized it less efficiently compared to males (Català-Niell et al., 2008). Furthermore, a previous study found that sex differences influence GLP-1 secretion differently (Handgraaf and Philippe, 2019). Testosterone can influence GLP-1 release in men, whereas estrogen and progesterone influence GLP-1 release in women (Handgraaf and Philippe, 2019). The difference in GLP-1 levels between normal male and female patients is due to the inconsistent glycemic control pattern between sexes (Pérez-Durillo et al., 2018). Notably, no significant findings for the FBS levels were observed between the HFD-fed and control groups. However, a significant difference between the 10SM and 10HM groups was observed in the pubertal period at approximately 8 weeks of age. HFD exposure at specific developmental times affects the blood sugar levels in male and female mice differently (Glavas et al., 2021). The effect of transient hyperglycemia observed in the 10HM group could be an early sign of adult metabolic syndrome due to developmental programming caused by HFD exposure during the pre- and post-natal periods in our study. A previous study determined that the effects of maternal overnutrition during the pre- or post-natal periods caused the offspring to develop metabolic syndrome and obesity (Padmanabhan et al., 2016). Although a protective effect was found in female mice, feeding male mice with HFD during the post-pubertal period led to an increase in diabetes mellitus risk (Morselli et al., 2014; Glavas et al., 2021). Conversely, another study has reported that the puberty period is associated with transient insulin resistance and the risk of type 2 diabetes mellitus in both male and female rats (Castell et al., 2022).

### 4.3 Reduced GIP mRNA expression in 10HM

The GIP mRNA expression in 10HM was significantly lower than that in 10HF. A previous study suggested that sex may influence the rate of gastric emptying time in response to food stimuli (Kautzky-Willer et al., 2016). Because fat slows down gastric emptying (Little and Feinle-Bisset, 2011; Warrilow et al., 2019), HFD in our study could potentiate this sex effect on the gastric emptying time. Furthermore, GIP expression in the rat SMG decreases with age (Privatananupunt et al., 2014). We observed that the mean GIP expressions in male and female rats at 10 weeks was lower than that in male and female rats at 3 weeks. This finding is congruent with a previous study, suggesting a possible association between GIP function and salivary gland development (Privatananupunt et al., 2014). Therefore, as the salivary gland becomes fully developed, the GIP level decreases (Privatananupunt et al., 2014). The small amount of GIP mRNA levels in the SMG of male and female rats at 10 weeks could be the reason why no significant changes were observed after HFD feeding in the 10HM and 10HF groups. As previously stated, the female GIP response to HFD may have been slower compared with that of the male group; therefore, a greater amount was identified in the female SMG.

### 4.4 HFD affects the SMG

Although the wet weights of SMG in 3-week-old rats fed on a HFD were slightly heavier than those of the standard diet groups in male and female rats, the difference was not significant. Several investigations found that, amongst all salivary glands, only the weight of the parotid glands between HFD-fed and control rats was significantly different (Kołodziej et al., 2017; Żukowski et al., 2018; Zalewska et al., 2019). However, the 12 weeks of HFD exposure in the 10HM group in our study covered all the phases of salivary gland development. Therefore, the wet weight of 10HM SMG was significantly heavier than that of 10SM. This finding is supported by a study which showed that after 6 weeks, rats fed on a HFD had significantly larger parotid glands and heavier SMG weights than control rats (Zalewska et al., 2020). Furthermore, intra-cytoplasmic vacuolations were identified in the SMG ductal cells in the HFD groups. Previous studies on HFD-fed rats have also reported lipid accumulation in the salivary glands (Kołodziej et al., 2017; Kandeel and M Elwan, 2023). These findings suggest that degenerative changes develop within the cells of the duct (Kandeel and M Elwan, 2023). The enlargement of the salivary gland caused by fat accumulation in patients with obesity or diabetes may result in salivary gland inflammation and dysfunction (Kołodziej et al., 2017).

### 4.5 Limitations and prospects

The remarkably low levels of GIP and GLP-1 mRNA expression reported in SMGs are one of our study’s limitations. Furthermore, the mRNA expression of GLP-1 and GIP was measured only in the gland, and the serum level was not determined. Moreover, the expression of GLP-1 was limited to only the salivary glands of rodents. Despite these limitations, this is the first study to quantify GLP-1 and GIP mRNA expression in the salivary gland under the HFD condition during pre- and post-natal periods. Furthermore, we also examined the influence of sex on GLP-1 and GIP. Our findings support the concept that long-term HFD changes GLP-1 mRNA expression. In the future, a more thorough investigation of the mechanism of action of incretin hormones in the SMG should be performed.

## 5 Conclusion

HFD, from maternal nutrition and environmental exposure insults throughout the pre- and post-natal periods, has been shown to reprogram the epigenome, contributing to the development of diseases later in life. The HFD exposure increased GLP-1 mRNA expression in SMGs of male rats after weaning and subsequent HFD feeding. In contrast, a decrease in GIP expression following the HFD was noted only in the young male rats. Although detected in a considerably low amount, the decreasing trend of GIP expression was observed subsequently after HFD feeding. Sex influences incretin hormones secretion and obesity-related conditions. Moreover, the physiological adaptations to HFD, such as obesity, the early stage of hyperglycemia, and the alteration of the salivary gland both in size and microstructure should be further evaluated. These findings indicate that there are developmental programming pathways and warrant intervention to prevent further metabolic diseases. Finally, our study lays a path for future investigations on the mechanism of action and function of the incretin hormones in the salivary glands, as well as the developmental programming pathways and roles of early detection and prevention.

## Data Availability

The datasets presented in this study can be found in online repositories. The names of the repository/repositories and accession number(s) can be found in the article/Supplementary Material.

## Abbreviations

3SM, 3-week-old male rats fed on a standard diet; 3HM, 3-week-old male rats fed on a high-fat diet; 3SF, 3-week-old female rats fed on a standard diet; 3HF, 3-week-old female rats fed on a high-fat diet; 10SM, 10-week-old male rats fed on a standard diet; 10HM, 10-week-old male rats fed on a high-fat diet; 10SF, 10-week-old female rats fed on a standard diet; 10HF, 10-week-old female rats fed on a high-fat diet; BSA, bovine serum albumin; BW, body weight; DAB, 3,3’- diaminobenzidine; DoHaD, Developmental Origins of Health and Disease; FBS, fasting blood sugar; GIP, glucose insulinotropic polypeptide; GLP-1, glucagon-like peptide-1; HFD, high-fat diet; OEA, oleoylethanolamine; PBST, phosphate-buffered saline with Tween^®^ 20; PH, pregnant rats fed on a high-fat diet; PS, pregnant rats fed on a standard diet; SMG, submandibular gland.
