# Supplementary material for: Expression of glucagon-like peptide-1 and glucose-dependent insulinotropic polypeptide in the rat submandibular gland is influenced by pre- and post-natal high-fat diet exposure
Source: Front Physiol. 2024 Mar 26;15:1357730. doi: 10.3389/fphys.2024.1357730 (PMC11002158; doi:10.3389/fphys.2024.1357730)
Supplement: Supplementary file 1 [file DataSheet1.pdf]

Supplementary Material

Expression of glucagon-like peptide-1 and glucose-dependent insulinotropic polypeptide in the rat submandibular gland is influenced by pre- and post-natal high-fat diet exposure

Pornchanok Sangsuriyothai[1, 2], Ippei Watari[1*], Saranya Serirukchutarungsee[1,3] Sirichom Satrawaha[2], Katarzyna Anna Podyma-Inoue[4], Takashi Ono[1]

[1]Department of Orthodontic Science, Graduate School of Medical and Dental Sciences, Tokyo Medical and Dental University (TMDU), Tokyo, Japan

[2]Department of Orthodontics, Faculty of Dentistry, Chulalongkorn University, Bangkok, Thailand

[3]Department of Pedodontics and Preventive Dentistry, Faculty of Dentistry, Srinakharinwirot University, Bangkok, Thailand

[4]Department of Biochemistry, Graduate School of Medical and Dental Sciences, Tokyo Medical and Dental University (TMDU), Tokyo, Japan

*** Correspondence:** Ippei Watari: ippeiwatari@gmail.com

1 Supplementary Table 1

Composition / Type of food	CE-2	HFD32
Moisture	9.05	6.20
Crude protein	24.80	25.50
Crude fat	4.60	32.00
Crude fiber	4.65	2.90
Crude ash	7.00	4.00
NFE	49.90	29.40

All values are reported as percentages by weight.

Supplementary Table 1 The composition of each type of food which were given to rats (the nutrient components in 100 g)
